# Significance of atherosclerotic plaque location in recanalizing non-acute long-segment occlusion of the internal carotid artery

**DOI:** 10.1038/s41598-024-61938-y

**Published:** 2024-05-13

**Authors:** Tong-Yuan Zhao, Gang-Qin Xu, Jiang-Yu Xue, Wei-Xing Bai, Dong-Yang Cai, Bo-Wen Yang, Wei-Yu Shi, Tian-Xiao Li, Bu-Lang Gao

**Affiliations:** Stroke Center, Cerebrovascular Disease Hospital, Henan Provincial People’s Hospital, 7 Weiwu Road, Zhengzhou, 450003 Henan Province China

**Keywords:** Hybrid operation, Atherosclerotic plaque, Long segment occlusion, Internal carotid artery, Complications, Diseases, Health care, Medical research, Neurology

## Abstract

To investigate the significance of atherosclerotic plaque location in hybrid surgery comprising both endovascular recanalization approaches and carotid endarterectomy for symptomatic atherosclerotic non-acute long-segment occlusion of the internal carotid artery (ICA), 162 patients were enrolled, including 120 (74.1%) patients in the proximal plaque group and 42 (25.9%) in the distal plaque group. Surgical recanalization was performed in all patients, with successful recanalization in 119 (99.2%) patients in the proximal and 39 (92.9%) in the distal plaque group. The total successful recanalization rate was 97.5% (158/162) with a failure rate of 2.5% (4/162). Periprocedural complications occurred in 5 (4.2% or 5/120) patients in the proximal plaque group, including neck infection in two (1.7%), recurrent nerve injury in 1 (0.8%), and laryngeal edema in 2 (1.7%), and 2 (4.8%) in the distal plaque group, including femoral puncture infection in 2 (4.8%). No severe complications occurred in either group. Univariate analysis showed plaque location was a significant (*P* = 0.018) risk factor for successful recanalization, and multivariate analysis indicated that the plaque location remained a significant independent risk factor for recanalization success (*P* = 0.017). In follow-up 6–48 months after the recanalization surgery, reocclusion occurred in two (2.8%) patients in the proximal plaque group and 4 (13.3%) in the distal plaque group. In conclusion, although hybrid surgery achieves similar outcomes in patients with ICA occlusion caused by either proximal or distal atherosclerotic plaques, plaque location may be a significant risk factor for successful recanalization of symptomatic non-acute long-segment ICA occlusion.

## Introduction

Non-acute occlusion of internal carotid artery (ICA) is usually caused by chronic development of atherosclerosis^1–6^, and chronic atherosclerotic occlusion of large intracranial arteries, including the ICA^5,7,8^, is one of the critical causes for ischemic cerebral stroke, accounting for approximately 15,000–20,000 ischemic events in the United States of America^9,10^. In the chronic process of stenosis progression, collateral arterial branches are often formed, and adjacent small vessels may also compensate, which improves the cerebral tissue perfusion. Therefore, after chronic arterial occlusion, a large amount of brain tissue can be compensated through the acute phase by collateral circulation of blood flow^8,11–13^. In the non-acute phase, the compensatory potential to the ischemic area may be impaired, with cerebral perfusion insufficiency and thrombus growth, leading to neurodegeneration and cerebral infarction. Studies have shown that the annual incidence of ipsilateral ischemic stroke in patients with chronic carotid artery occlusion receiving drug treatment is 3%^14^ but will increase to 10%–20%^15^ if combined with severe cerebral hemodynamic disorders. Bypass surgery may not reduce the ipsilateral recurrent stroke rate two years later^16^. Endovascular recanalization and hybrid surgery comprising endovascular approaches and carotid endarterectomy (CEA) have been applied for the treatment of non-acute ICA occlusion, leading to good effects^1–6,17–21^. For long-segment ICA occlusion involving the initial ICA C1 segment^22^, CEA is good to eliminate the carotid plaques before endovascular treatment for intracranial lesions. For atherosclerotic long-segment ICA occlusion without involvement of the initial ICA C1 segment, endovascular treatment is needed, however, a neck cut and an arteriotomy on the carotid artery are still necessary to remove the atherosclerotic plaque, which is similar to the CEA procedure. In these circumstances, the location of the atherosclerotic plaque may be important in the performance and efficiency of the hybrid surgery in the treatment of symptomatic atherosclerotic long-segment occlusion of the ICA. It was hypothesized that the location of atherosclerotic plaques might affect successful hybrid surgical recanalization of symptomatic chronic long-segment ICA occlusion, and the purpose of this study was to test this hypothesis.

## Materials and methods

**Figure 1:**
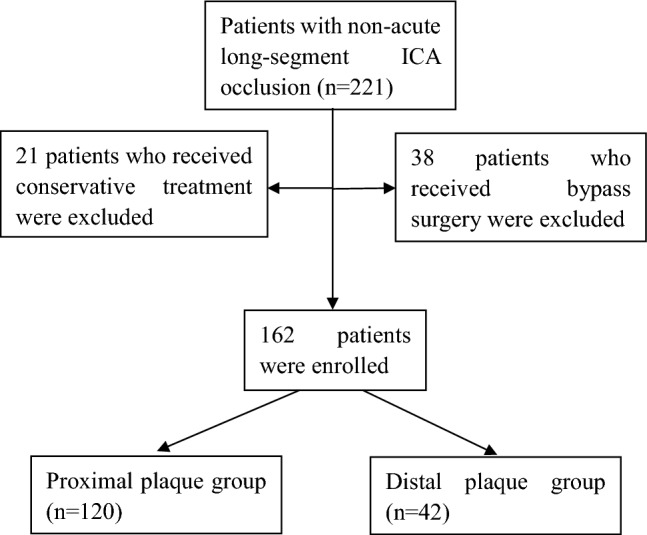
The flow chart of patient enrollment.

This single-center retrospective study was approved by the ethics committee of Henan Provincial People’s Hospital, and written informed consent was waived by the same ethics committee because of the retrospective study design. All methods were performed in accordance with the relevant guidelines and regulations. Between January 2014 and January 2023, patients with non-acute (> 7 days) atherosclerotic ICA long-segment occlusion treated with hybrid surgery comprising endovascular recanalization and CEA or arteriotomy of the carotid artery to remove the plaque were retrospectively enrolled (Fig. 1). The inclusion criteria were patients with non-acute long-segment occlusion of the ICA confirmed by digital subtraction angiography, detection of the atherosclerotic plaque location by color ultrasound or high-resolution magnetic resonance imaging (MRI) and 3D-TOF MRI, progression or aggravation of the disease according to imaging examination, increase of the National Institutes of Health Stroke scale (NIHSS) over 4 scores or the modified Rankin Scale (mRS) score over 1 score, over two ICA anatomical segments being occluded or ≥ 6 cm in length or in tandem occlusion, and treatment of hybrid surgery. The exclusion criteria were patients with acute ICA occlusion, short occlusion less than two segments, radiation therapy history of neck diseases with bilateral pharyngeal muscle paralysis, history of subarachnoid hemorrhage, severe systematic diseases, and intolerant to the hybrid surgery. Based on the ICA classification of seven segments from C1 to C7 in an anterograde direction (C1, cervical; C2, petrous; C3, lacerum; C4 cavernous; C5, clinoid; C6, ophthalmic; and C7, communicating)^22^, the patients enrolled were assigned into two groups: proximal plaque group with the atherosclerotic plaque involving the ICA initial C1 segment and distal plaque group with the atherosclerotic plaque located distal to the ICA C1 segment. The proximal plaque group was treated with CEA in the first place before endovascular recanalization of distal intracranial lesions, whereas the distal plaque group was treated with a neck cut and an arteriotomy on the carotid artery before inserting endovascular devices through the arteriotomy to recanalize the atherosclerotic lesion.

### Hybrid surgical procedures

Before the surgery, imaging was performed to confirm the occluded lesion responsible for all the relevant symptoms. Dual antiplatelet therapy was administered to every patient using clopidogrel (75 mg/d) and aspirin (100 mg/d). Tirofiban was injected intravenously at the dose of 10 μg/kg within 3 min, and later, dripped intravenously at the dose of 0.15 μg/kg for 36–48 h. Two hours before the surgery, nimotone was pumped intravenously, and atorvastatin (20 mg/d) was administered at the dose of 20 mg/d for those with normal hepatic and renal function.

The hybrid surgery was conducted under general anesthesia, and systematic heparain was administered based on the body weight with 3000 u-6000 u injected intravenously, and additional 800 u–1000 u was added every hour to maintain the activated clot time of 250–300 s. After femoral access, a 6F femoral arterial sheath was inserted before a 6F MPD guiding catheter (Cordis, USA) was navigated to the diseased artery. This pathway was used for cerebral angiography, balloon dilation and/or stent deployment after plaque removal through the CEA or carotid arteriotomy.

For proximal plaques, CEA was performed after angiography confirmed the location of the plaque. A cut was conducted along the anterior margin of the sternocleidomastoid muscle to expose the carotid bifurcation before a longitudinal arteriotomy on the common and internal carotid arteries to remove the atherosclerotic plaque. Under direct view, a Progreat guide wire (0.021″) plus a micro-catheter (0.025″, Telmo, Japan) was introduced via the arteriotomy to pass carefully through the thrombus location of the ICA before withdrawing the guide wire. A Traxcess-14 (Microvention, USA) 200-cm-long micro-guide wire was introduced within the micro-catheter to the straight M1 or M2 segment of the middle cerebral artery before a Traxcess-14 (Microvention, USA) 115-cm-long guide wire was exchanged with the Progreat micro-catheter. A Fogarty 2F or 3F thrombectomy catheter was introduced along the Traxcess micro-guide wire to the ICA cavernous segment before the balloon was inflated for thrombectomy. After thrombectomy, the thrombi were examined for the wholeness, and if necessary, a micro-catheter was introduced for angiography to confirm residual of thrombi. Stent deployment was conducted with an appropriate stent being selected with its diameter being 1 mm less than that of the proximal ICA before the arteriotomy and neck cut were closed.

For distal plaques, CEA was not necessary because the atherosclerotic plaques were located distal to the ICA C1 segment, however, a neck cut and an arteriotomy were still needed to remove the atherosclerotic plaque. Endovascular treatment was performed with the Progreat guide wire and catheter being navigated via the MPD guide catheter to a place distal to the ICA thrombi. In the proximal segment, the thrombi were soft and loose and were easily passed through, whereas in the distal segment, the thrombi were hardened and not readily passed across, which necessitated use of an Echelon-10 micro-catheter or a SL-10 micro-catheter in combination with a Traxcess-14 or a Asahi-14 micro-guide wire to recanalize the occluded lesion. During recanalization of the lesion, the micro-guide wire was rotated and navigated along the potential space within the plaque to recanalize the artery. If the guide wire and micro-catheter could not be navigated across the occluded location, endovascular operation was stopped to prevent further injury to the arteries. After the catheter and micro-guide wire reached the real arterial lumen distal to the occlusion, the micro-guide wire tip was put at the M1 or M2 straight segment of the middle cerebral artery before withdrawing the micro-catheter. Then, a longitudinal cut was conducted along the sternocleidomastoid muscle anterior margin to expose the carotid bifurcation before a longitudinal arteriotomy on the ICA to retract the proximal end of the micro-guide wire. Under fluoroscopy, a Fogarty balloon was used for thrombectomy before closure of the arteriotomy and the neck cut. Then, endovascular operation was performed using the MPD guide catheter. Because the micro-catheter and the micro-guide wire had been put distal to the occlusion, it was easy to pass through the occluded segment, and angiography was performed for necessary endovascular treatment including balloon dilation and stent deployment. An appropriate stent was selected with its diameter being 1 mm less than that of the proximal ICA and deployed at the occluded location after balloon angioplasty.

### Assessment.

According to the thrombolysis in cerebral infarction (TICI) grading standards^23^, the blood flow through the recanalized artery was evaluated, and recanalization was considered successful if TICI ≥ 2b. After the surgery, head computed tomography (CT) was conducted to exclude possible intracranial hemorrhage, and head CT was repeated 3–6 h for patients with suspected intracranial hemorrhage. MRI was performed 48 h later to show if de novo intracranial infarction existed.

### Statistical analysis

The statistical analysis was conducted in this study with the SPSS software (version21.0, Chicago, IL, USA). Continuous measurement data were presented in the mean and standard deviation if in the normal distribution and tested with the t test or in the median and interquartile range if not in the normal distribution and tested with the Mann Whitney U test. Categorical data were presented in frequency and percentage and tested with the Chi square test. Univariate logistic regression analysis of risk factors associated with complications and successful recanalization in the total group with the two groups put together, including age, sex, mRS, location, hypertension, diabetes mellitus, smoking, alcohol abuse, cardiac diseases, number of past infarction, duration to the recanalization surgery, collateral circulation, and use of CEA. Multivariate logistic regression analysis for independent risk factors was performed using significant factors in the univariate logistic analysis. The significant *P* value was set at *P* < 0.05.

## Results

A total of 162 patients were enrolled (Table 1), including 120 (74.1%) patients in the proximal plaque group and 42 (25.9%) in the distal plaque group. No significant (*P* > 0.05) difference was found in age, sex components, mRS, hypertension, diabetes mellitus, smoking, alcohol abuse, past infarction history, heart disease, and duration from disease onset to surgical recanalization between the two groups (Table 1).

**Table 1:** Demography of patients in two groups.

Variables	Proximal plaque group (120)	Distal plaque group (42)	*P*
Sex (M/F)	88/32	25/17	0.35
Age (years)	35–71 (53.9 ± 9.2)	42–75 (55.2 ± 9.9)	0.52
mRS	1–4 (2.45 ± 0.90)	1–4 (2.56 ± 0.82)	0.47
Hypertension (n, %)	65 (54.2%)	24 (57.1%)	0.52
Diabetes mellitus	32 (26.7%)	10 (23.8%)	
Smoking	75 (62.5%)	28 (66.7%)	
Alcohol abuse	26 (21.7%)	12 (28.6%)	
Past infarction	19 (15.8%)	6 (14.3%)	
Heart disease	27 (22.5%)	9 (21.4%)	
Duration to surgery (d)	17–360 (30, 25–44)	19–330 (28, 32–46)	0.42

**Figure 2:**
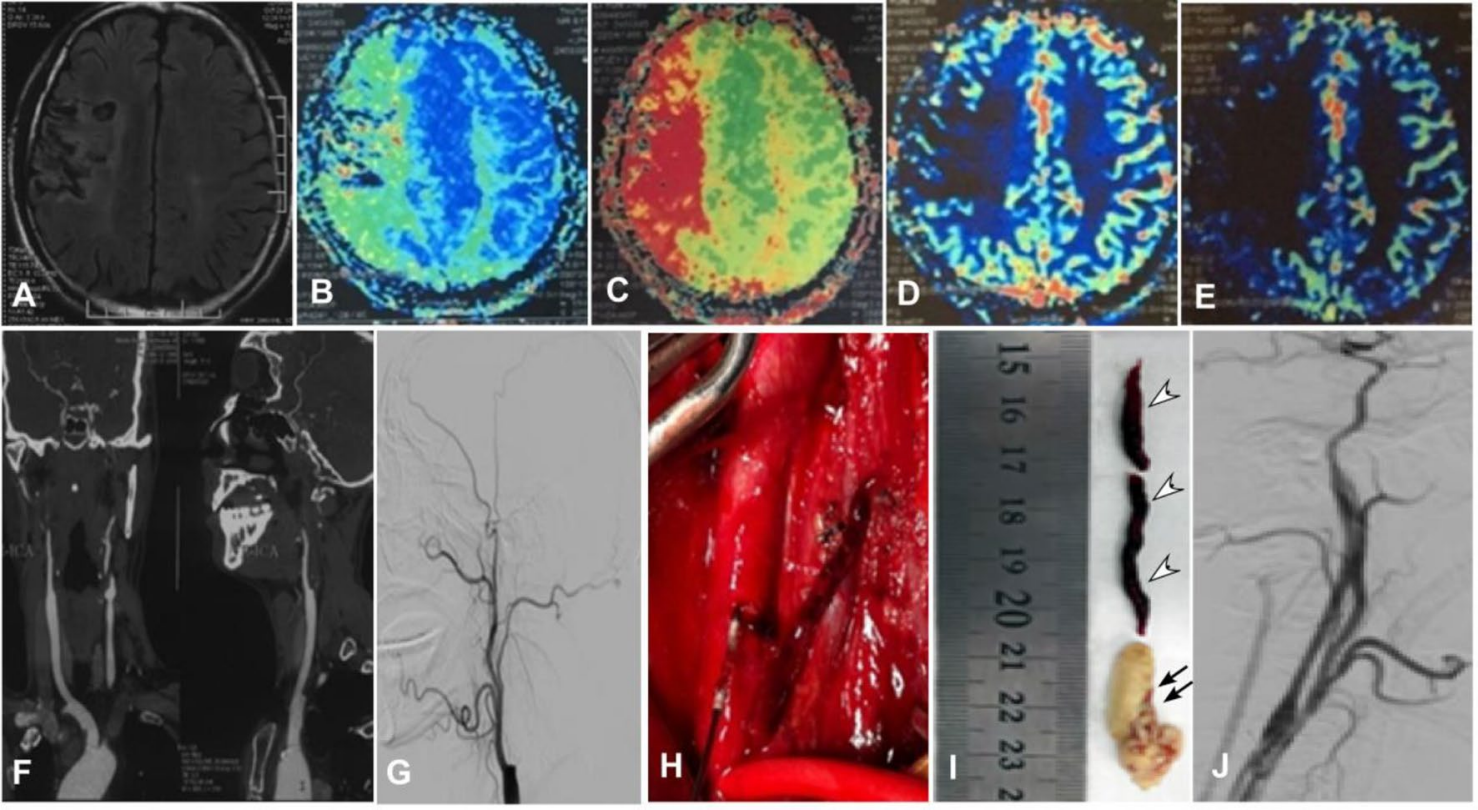
Proximal atherosclerotic plaques involving the common carotid artery bifurcation and causing long-segment occlusion of the right internal carotid artery (ICA) treated with hybrid surgery comprising carotid endarterectomy (CEA) and endovascular treatment in a patient in their 50 s. (**A**) Magnetic resonance imaging (MRI) showed multiple infarcts in the right semioval region. (**B**–**E**) The time-to-peak (**B**) and mean transit time (**C**) were prolonged, and the cerebral blood volume (**D**) and cerebral blood flow (**E**) were decreased. (**F**) Computed tomography angiography revealed occlusion of the right ICA. (**G**) Digital subtraction angiography demonstrated occlusion of the right ICA. (**H**) CEA was conducted, and a Forgarty balloon was used for removal of the atherosclerotic plaque. (**I**) The atherosclerotic plaque (double arrows) and the thrombi (arrow heads) were approximately 8 cm long. (**J**) After the surgery, the ICA occlusion was successfully recanalized.

**Figure 3:**
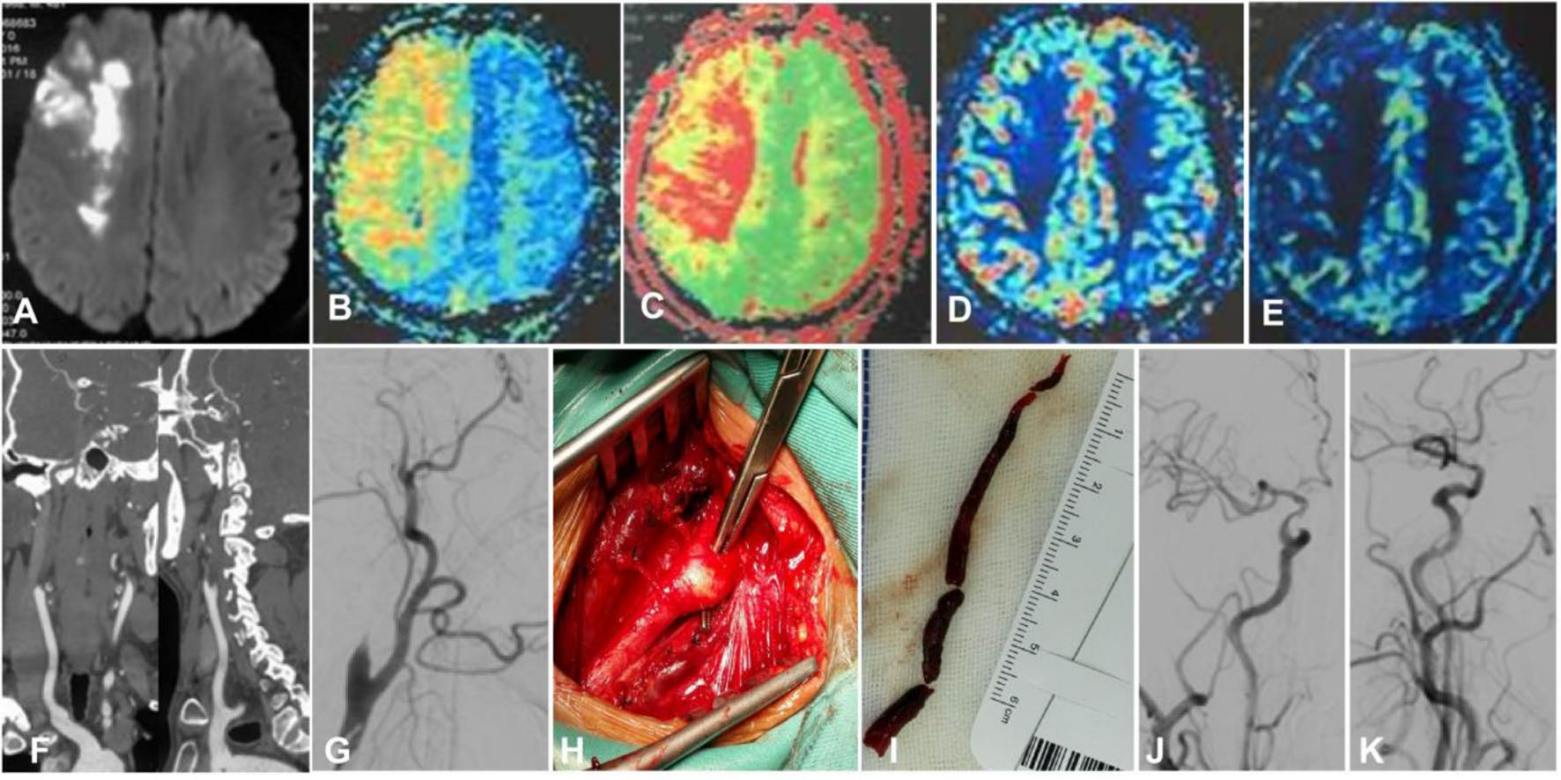
Distal atherosclerotic plaques causing long-segment occlusion of the internal carotid artery (ICA) treated with hybrid surgery in a patient in their 40 s with dizziness and left limb weakness. (**A**) Magnetic resonance imaging (MRI) showed multiple acute infarction lesions in the right hemisphere. (**B**–**E**) The time-to-peak (**B**) and mean transit time (**C**) were prolonged, and the cerebral blood volume (**D**) and cerebral blood flow (**E**) were decreased. (**F**) Computed tomography angiography revealed occlusion of the right ICA. (**G**) Digital subtraction angiography demonstrated occlusion of the right ICA. (**H**,**I**) Arteriotomy of the right ICA was performed for use of a Forgarty balloon to remove the atherosclerotic plaque (**H**), and the thrombus was approximately 7 cm long (**I**). (**J**,**K**) After plaque removal and recanalization of the ICA, a stenosis of the ICA ophthalmic segment was treated with balloon dilation, and smooth blood flow was resumed in the right ICA even though a slight stenosis remained at the ICA ophthalmic segment.

Surgical recanalization was performed in all patients (Figs. 2, 3), with successful recanalization in 119 (99.2%) patients in the proximal plaque group and 39 (92.9%) in the distal plaque group, with no significant (*P* = 0.52) difference between the two groups. One (0.8%) patient was not successfully recanalized in the proximal group and 3 (7.1%) in the distal group. The total successful recanalization rate was 97.5% (158/162) with a failure rate of 2.5% (4/162).

Periprocedural complications occurred in 5 (4.2% or 5/120) patients in the proximal plaque group, including neck infection in two (1.7%), recurrent nerve injury in 1 (0.8%), and laryngeal edema in 2 (1.7%), and 2 (4.8%) in the distal plaque group, including femoral puncture infection in 2 (4.8%). There was no significant difference in the complication rate between the two groups. No severe complications occurred like cerebral infarction, hemorrhage, neurological deficits, or death in either group.

Univariate analysis of factors associated with complications and successful recanalization in the total group with the two groups put together, including age, sex, mRS, location, hypertension, diabetes mellitus, smoking, alcohol abuse, cardiac diseases, number of past infarction, duration to the recanalization surgery, collateral circulation, and use of CEA. Successful recanalization was significantly associated with plaque location (Chi square 5.56, *P* = 0.018). No significant (*P* > 0.05) factors were associated with periprocedural complications. Multivariate analysis of factors (plaque location, mRS, duration to the surgical recanalization, and use of CEA) for independent risk factors for successful recanalization in the total group revealed that the plaque location remained a significant independent risk factor for recanalization success (*P* = 0.017).

Follow-up was performed 6–48 (median 25) months after the recanalization surgery, including 72 (60%) patients in the proximal plaque group and 30 (71.4%) in the distal plaque group. Reocclusion of the original occluded ICA was present in two (2.8%) patients in the proximal plaque group and 4 (13.3%) in the distal plaque group, with no significant (*P* > 0.05) difference between the two groups (Table 2).

**Table 2:** Surgical outcomes and follow-up.

Variables	Proximal plaque group (120)	Distal plaque group (42)	*P*
Successful recanalization	119 (99.2%)	39 (92.9%)	0.52
Failed recanalization	1 (0.8%)	3 (7.1%)	
Complications	5 (4.2%)	2 (4.8%)	0.23
Puncture infection	2 (1.7%)	2 (4.8%)	
Recurrent nerve injury	1 (0.8%)	0	
Laryngeal edema	2(1.7%)	0	
Follow-up			
Time (m)	6–42 (14 ± 6)	6–48 (16 ± 9)	
Number of patients (n, %)	72 (60%)	30 (71.4%)	
Reocclusion	2 (2.8%)	4 (13.3%)	0.62

## Discussion

Our study investigated the significance of atherosclerotic plaque location in hybrid surgical treatment of symptomatic non-acute ICA long-segment atherosclerotic occlusion, and it was found that the location of atherosclerotic plaque was an important risk factor for successful recanalization of non-acute long-segment ICA occlusion using the hybrid surgical recanalization approach. Although the successful recanalization rate was greater in the proximal plaque group than in the distal plaque group (99.2% vs. 92.5%), there was no significant (*P* = 0.52) difference in the recanalization rate between the two groups.

Atherosclerosis is a chronic process, and development from arterial stenosis to complete occlusion will cause chronic low cerebral perfusion and subsequent formation of collateral circulation. Chronic ICA occlusion may cause no clinical symptoms in patients with good collateral circulation but may also result in strokes in patients with poor collateral circulation. Atherosclerosis is the most important factor to cause ICA occlusion^24^, with a high incidence of approximately 70%^25^, which is greater than that caused by dissection, arteritis, trauma, and radiation. Patients with ICA occlusion may have a high incidence of strokes, especially in patients with concurrent low cerebral perfusion^15^. For symptomatic ICA occlusion caused by low perfusion, medication is not efficient to decrease the long-term rate of stroke^26,27^. Intracranial and extracranial vascular bypass surgery may increase the intracranial perfusion but its effect may not be as good as medication^16^. The CEA is only suitable for patients with occlusion at the initial ICA segment^28^, and endovascular recanalization alone may recanalize long-segment occlusion but with a low success rate^29,30^. The hybrid surgery comprises CEA or carotid arteriotomy to remove the atherosclerotic plaque, balloon thrombectomy, balloon and stent angioplasty, which is good for recanalization of long-segment occlusion, high-location occlusion, occlusion involving the ICA initial segment, and tandem occlusion lesions.

After atherosclerotic occlusion of the ICA, thrombi may be formed and organized with time. Compared with the atherosclerotic plaques, thrombi formed at a later time are usually less fibrous and calcified. In endovascular recanalization, the micro-catheter and micro-guide wire may not easily pass through the atherosclerotic plaque compared with newly-formed thrombi. This is why further endovascular recanalization operation was stopped to prevent injury to the arterial wall if the guide wire and micro-catheter could not be successfully navigated through the occluded location. In this case, the lesion was probably composed of organized thrombi rich in fibrous tissue with a hard texture adhering to the intima of the arterial wall. Although we have tried forced suction with aspiration catheters, it does not help because of the organized thrombi rich in fibrous tissue to connect with the arterial wall. In recanalization of chronic ICA occlusion, the Fogarty balloon is better for thrombectomy because of its strong force compared with a stent retriever or an aspiration catheter. In addition, the intracranial ICA is tortuous, a guide wire may not be easily controlled when passing through the plaque, thus causing dissection, arterial perforation, and plaque fragmentation to form emboli and subsequent complications. In hybrid surgery, CEA is performed for proximal thrombi, which can avoid difficulty passing a guide wire through the plaque in mere endovascular operation. After thrombectomy with CEA, the thrombi within the ICA lumen are soft and loose and can be easily passed through by a guide wire. Nonetheless, for distal plaques, a guide wire and a catheter may not pass through the distal plaque even if a hybrid surgery can evacuate the proximal thrombi. In theory, hybrid surgery may not be better than mere endovascular treatment in successful recanalization of distal plaques, however, hybrid surgery can provide additional benefits than just recanalizing the occluded artery, with the benefits of eliminating intraarterial thrombi, increasing blood flow volume, and decreasing the risk of long-term restenosis or re-occlusion. In our study, the success recanalization rate and long-term reocclusion rate were better in patients with proximal plaques than those with distal plaques even though no significant differences were detected. Nonetheless, when the two groups of patients were put together, the atherosclerotic plaque location was detected to be a significant independent risk factor for successful hybrid surgical recanalization, which needs further confirmation.

For hybrid surgery to recanalize long-segment ICA occlusion, it is necessary to determine the exact location of plaques, which may be helpful for increasing the surgical success rate and decreasing the surgical risk. Currently, carotid ultrasound and high-resolution MRI are frequently used in this aspect. Carotid ultrasound is fast, easy and applied widely, being able to detect thrombi, plaques, intra-plaque hemorrhage, and completeness of the arterial wall in proximal ICA segments^28^. High-resolution MRI is able to effectively display arterial components, demonstrate the plaque size and range, and make quantitative and qualitative imaging of the occluded artery for both proximal and intracranial ICA plaques^31–34^. Nonetheless, 3D-TOF imaging may be needed for tortuous arteries with partial volume effect because the tortuous arteries may be regarded as thickened intima^35,36^.

Recently, more patients had been treated with hybrid surgery with good outcomes^6,37–40^. Because few medical centers are able to perform the hybrid surgery, no definitive surgical regulations and standards have been set up. Nonetheless, the determination of atherosclerotic plaque location is very important for successful surgery and decreased surgical risk.

Some limitations existed in our study, including the single-center and retrospective study design, no randomization, and a small cohort of patients, which may all affect the generalization of the outcomes. Moreover, the sample size of this study is small and does not meet the requirements of Event Per Variable, especially for patients who failed to be successfully recanalized. Therefore, the results may not be robust enough. However, considering that this type of patient was rare and the results had some interpretability, the multivariate analysis was still conducted to detect the possible independent risk factor even though further research is needed to confirm the reliability of this result. Future prospective, randomized, controlled studies with multiple centers and multiple races and ethnicities involved will have to be conducted to obtain better outcomes.

In conclusion, location of the atherosclerotic plaques may be an important factor to affect successful recanalization of chronic long-segment atherosclerotic occlusion of the internal carotid artery even though more stringent studies are necessary to prove this outcome.

## Data Availability

The datasets used and/or analysed during the current study available from the corresponding author on reasonable request.
